# Limited care offered to people with epilepsy in Mwanza, Tanzania: need for intervention

**DOI:** 10.11604/pamj.2021.38.407.28321

**Published:** 2021-04-29

**Authors:** Haruna Dika, Rahel Nkola, Shabani Iddi, Catherine Magwiza, Gilbert Kongola

**Affiliations:** 1 Catholic University of Health and Allied Sciences, Mwanza, Tanzania,; 2 Bugando Medical Center, Mwanza, Tanzania

**Keywords:** Epilepsy, socioeconomic, counseling, patient, Tanzania

## Abstract

**Introduction:**

epilepsy is a very common neurological disorder which is associated with high socioeconomic burden. While up to 90% of people with epilepsy (PWE) in developing countries do not get appropriate treatment, there is limited information about care offered to PWE in Tanzania. This study aimed to describe available care offered to PWE in Mwanza.

**Methods:**

a cross-sectional study involving health care workers (HCWs) and PWE attending five selected hospitals outpatient clinics of Mwanza region was done. HCWs completed self-administered questionnaires while PWE or caretakers were interviewed using structured questionnaires. Coded data were analyzed using SPSS.

**Results:**

a total of 18 HCWs and 218 PWE (or their care takers) participated in this study. Health care workers rarely used investigations to confirm epilepsy diagnosis or explore its causes. 10/18 (55.6%) of HCWs reported that counseling was given to patients but counseling information was largely inadequate regarding the use of anti-epileptic drugs (AEDs). The AEDs prescriptions were dictated by drug availability and affordability to patients. Among 197 PWE, whose AEDs doses were revealed, 136 (69.0%) were under-medicated. No follow-up investigation was done to all PWE who were interviewed. There was discrepancy between hospitals and practitioners regarding withdrawal of AEDs.

**Conclusion:**

people with epilepsy in Mwanza received limited care. Patients were not thoroughly investigated, counseled and followed-up, and had limited choice and accessibility to AEDs. Some patients particularly in district hospitals were under-medicated despite of seizure recurrence. We recommend short-course training about epilepsy management to the HCWs who diagnose and treat PWE regularly.

## Introduction

Epilepsy is a very common chronic neurological disorder affecting more than 50 million people worldwide, with majority being from developing countries [1,2]. Epilepsy is associated with high socioeconomic burden, which is due to increased health care cost as well as losses in employment, wages, and household work [3]. Successful treatments of epilepsy in a majority of patients depends on appropriate diagnosis, correct choice of anti-epileptic drugs (AEDs) and patient adherence to prescribed AEDs. In developing countries, reports showed that, up to 90% or more of people with epilepsy (PWE) were not getting appropriate treatment [4,5]. However, a relatively recent report from Tanzania showed that only 40.5% of PWE do not get treatment [6]. Tanzania has limited medical human resources and Tanzanians have low income, the factors which can greatly compromise health care delivery. However, there is limited information about how PWE who attend health facilities are managed; in terms of facilities arrangement, who attends them, diagnostic methods and treatment offered to them, and if follow-up is adequately done. Therefore, the aim of this study was to describe the available care offered to PWE in Mwanza region health facilities.

## Methods

**Study design:** a cross-sectional descriptive study was conducted to determine the available care offered to PWE.

**Setting:** the study was carried out from November 2016 to May 2017. It was conducted in five selected government hospitals of Mwanza region, in Tanzania. Mwanza is one of the regions in the Lake Zone. It is the region with the highest population in the Lake Zone. It has an estimated population of 2.8 million. One of the five hospitals where this study was carried out is Bugando Medical Center (BMC), which is a consultant referral hospital. Other four were district hospitals, namely Magu, Kwimba, Ukerewe and Sengerema. The four selected district hospitals are among seven district hospitals of Mwanza region. These hospitals were selected purposely because they are distant from BMC and therefore give general representation of the region. Two of the three district hospitals which were not selected, are 5-6 kilometres (km) from BMC and one is 49 km away. Patients suspected or diagnosed in these three hospitals were often referred to BMC for management. As per clinic records, about 180 PWE were regularly attended at BMC and a total of about 220 in the four selected district hospitals (Magu ~ 60, Kwimba ~ 40, Ukerewe - 48 and Sengerema ~ 70).

**Study participants:** participants in this study were health care workers (HCWs), who regularly diagnose and treat PWE in the five selected hospitals and PWE (or their caretakers) attending outpatient clinics at these hospitals.

**Sample size determination and sampling procedures:** due to small number of HCWs who regularly attend PWE, we aimed to enroll all HCWs who voluntarily agreed to participate in the study. The sample size for PWE was determined using Taro Yamane formula [7],

$$n=\frac{N}{1}+N{\left(e\right)}^{2}$$

where n = sample size, N = number of people in the population and e = allowable error (%). Using an estimated total number of 400 PWE attending the selected hospitals in Mwanza region and assuming allowable error of 0.05% and 95% confidence level, the minimum sample size was determined to be 200. A convenience sampling technique was used to enrol study participants.

**Data collection:** data from HCWs were collected using self-administered pretested structured questionnaires. We interviewed PWE (or their caretakers) using pretested structured swahili translated interview guides. In the event that a patient or caretaker was not competent in swahili, a third person of choice by the patient (either a relative or HCW) was used as a translator. The questionnaires and interviews aimed to determine the hospital arrangements for managing PWE, available facilities, who diagnosed and treated PWE, available/used AEDs, how AED treatments were initiated, instructions given to patients, basic investigations which were carried out, follow up of treatment and procedures on drugs discontinuation. In addition, dosages of used AEDs were judged as therapeutic or subtherapeutic based on dosages as indicated in the Tanzanian standard treatment guidelines.

**Data analysis:** data from HCWs and those from PWE (or their caretakers) were analysed separately. Qualitative data were coded. The data were double entered into Microsoft Excel data sheets, cross checked and then transferred to SPSS for Windows version 20 (SPSS, Atlanta, GA, USA) for analysis. Descriptive statistics were carried out to determine patients´ ages mean ± standard deviation and coded qualitative data frequencies and percentages. Results are summarized in tables.

**Ethical considerations:** ethical clearance and approval was obtained from Joint Catholic University of Health and Allied Sciences and BMC Research and Ethical Committee under reference number CREC/120/2016. Permission to conduct the study was obtained from Mwanza regional Commissioner´s office, Bugando Medical Center and respective District hospitals administrations. All study participants signed consents after adequately be informed about the objective of the study, the right not to participate in the study or withdrawal from the study at any time they want. All information obtained in the study was kept confidential.

## Results

### Characteristics of the study participants

**Characteristics of interviewed health care workers:** a total of 18 (8 males, 10 females) out of 21 HCWs who routinely diagnosed and treated PWE in Mwanza region were enrolled in this study. The largest number (8; 44.4%) of them were nurses Table 1. There were no epilepsy specialists (neurologists) in the region (Table 1).

**Table 1:** Interviewed health care workers who diagnosed and treated PWE

Cadre	Number	Percentage
Psychiatrist	1	5.6
General Medical Officers	5	27.8
Clinical officers	4	22.2
Nurses	8	44.4
**Total**	18	100

**Characteristics of PWE participants:** a total of 218 PWE (106 males, 112 females) with mean age of 29.0 ± 13.7 years were enrolled in this study. Among 218 PWE, 56 (25.7%) were recruited from BMC (consultant referral hospital) and others were from district hospitals (Sengerema- 50 (22.9%), Magu- 40 (18.3%), Ukerewe- 40 (18.3%) and Kwimba- 32 (14.7%).

**Hospital units for managing people with epilepsy:** in all five hospitals, there were no special units for managing PWE. In 4/5 hospitals PWE were treated in psychiatric or mental health clinics, while in the remaining one hospital they were managed in general outpatient clinic. Electroencephalogram (EEG), computed tomography (CT) scan and AED level monitoring facilities were available only in one centre (consultant referral hospital). Magnetic resonance imaging (MRI) machine was not available in all five hospitals. Electrocardiogram (ECG) machines were available in the consultant referral hospital and one district hospital while echocardiogram was found only in the consultant referral hospital. Facilities for measuring hematological profile, serum electrolytes and liver functions were available in all five hospitals.

**Documentation of epilepsy cases:** in all five centres, epilepsy cases were documented without seizure classification.

**Investigations and treatment of epilepsy:** only 2/18 (11.1%) HCWs, all from the consultant hospital, reported that they sometimes used investigations to confirm epilepsy diagnosis or explore its causes. Out of 218 PWE, only 11 (5.0 %), 3 (1.4%), and 1 (0.5%) had EEG, brain CT scan and brain MRI scan done to them. None of the patient had cardiac investigations (ECG or echocardiograph) done to exclude cardiac syncope or fainting from epileptic seizures. No follow-up investigations were done in any of the 218 participant patients. Out of 18 HCWs, 10 (55.6%) reported that counseling was given to newly diagnosed PWE. On other hand, 160/218 PWE or caretakers (73.4%) reported that, discussions about what epilepsy means, how is it acquired and how can it be treated were given to them. One hundred sixty-seven (76.6%) PWE were told about the importance of using AEDs and 75 (34.4%) were informed about the available AEDs to use and their costs. Only 34/218 patients (15.6%) were told about the AEDs side effects. While 140/218 PWE (64.2%) were told to avoid alcohol use, only 7 (3.2%) of them were told about the drugs to avoid while using AEDs. Phenytoin (PHT) and phenobarbital (PB) were mostly used AEDs. In Magu district hospital, 38/40 patients used PHT and/or PB; one used carbamazepine (CBZ) and one used PHT and CBZ. In Kwimba district hospital, all patients used PB, with four using either PHT or CBZ in addition. In Sengerema district hospital, 41/50 patients used PHT and PB together, 6/50 used PB alone, 2/50 used PHT and CBZ and 1/50 was not on AED. In Ukerewe district hospital, 31/32 PWE were on PHT, PB and CBZ either as monotherapy or in combination and one used levetiracetam in combination with PB. Fifty-six patients treated at BMC used PHT, PB, CBZ or valproic acid (VPA) either as monotherapy or combination of two drugs. The AEDs prescriptions were mainly dictated by drug availability and affordability to patients (Table 2). In district hospitals, sometimes patients were given a different AED in each outpatient visit, depending on which drug was available on that day. Four patients (1.8%) were found to have used the drugs that are normally contraindicated to use with AEDs. Twenty-seven out of 218 (12.4%) and 109/218 (50.0%) patients were given sub-therapeutic and sub-optimal therapeutic doses respectively (Table 3). Among the patients who were given sub-therapeutic and sub-optimal therapeutic doses, 15 (55.6%) and 57 (52.3%) respectively had one or more seizures in the past 30 days. Ten out of 18 (55.6%) prescribers did not stop AED medications in their patients despite of long seizure-free periods, and the rest stopped medications after varying periods (1-5 years) of being seizure-free.

**Table 2:** choice of AEDs for prescription

Reason for prescription	Number of practitioners (n=18)	Percentage of practitioners
Affordability to patients	14	77.8
Availability of drug in the hospital or market	18	100.0
AED side effects	7	38.9
Seizure type	3	16.7
Other reasons	4	22.2

* Respondents gave multiple reasons

**Table 3:** dosages of AEDs used by PWE treated in Mwanza

Dosage of AEDs used	Frequency (and percentage)					
	Magu	Kwimba	Sengerema	Ukerewe	BMC	Total
Sub-therapeutic	6	1	1	16	3	27
Sub-optimal therapeutic	33	1	32	9	34	109
Optimal therapeutic		36	3	6	16	61
**Total**	**39**	**38**	**36**	**31**	**53**	**197**

* In 21 patients, doses were not documented or patients were not on medication

## Discussion

This is one of few studies in Africa to describe the available health care, which is offered to PWE in terms of management setting, diagnosis, counseling, treatment and follow up. Lack of epilepsy/neurology clinics in Mwanza might be due to lack of epilepsy specialists in the region to run the clinics. There are seven practicing neurologists in Tanzania, but none is based in Mwanza and Lake Zone regions. Six of them are based in Dar-es-Salaam and one in Moshi. Lack of neurologists in the lake zone regions and other areas of the country has led PWE to be managed in psychiatric or mental health clinics. Due to lack of neurologists in Mwanza, PWE were diagnosed and treated by nurses, general doctors and clinical officers. This is contrary to earlier report, which showed that in more than 55% of low-income countries, epilepsy specialists (neurologists) were available to provide care to PWE [8]. All patients who are suspected to have epileptic seizures are recommended to be seen by a neurologist or other specialist for accurate diagnosis and optimal management [9,10]. Epilepsy specialists have a role of diagnosing and documenting the cases of epilepsy, performing investigations as well as treating and following up of epileptic patients. Epilepsy specialists also do provide education services and counseling to PWE and general public. Although failure of suspected new epilepsy cases to be seen by a neurologist is not a new phenomenon [11], PWE need to be evaluated by a higher level trained and experienced clinician. Lower cadre health workers are likely to lead to a number of misdiagnosis [12]. This might lead to unnecessary or delayed use of AEDs. Although no one can completely avoid misdiagnosis, neurologists can make the most accurate epilepsy diagnosis compared to nurses, general doctors or other specialists. In some cases, a neurologist´s opinion is most important in arriving into correct diagnosis of epilepsy. For instance, in the United Kingdom it was found that neurologist final diagnosis of suspected new epilepsy significantly differed from that of specialist registrars [11]. While missing the diagnosis of genuine epilepsy only delays initiation of AEDs, a false epilepsy diagnosis may have severe psychological and socioeconomic consequences for the patient and the family in general. Inadequacy of HCWs trained in diagnosing and treating epilepsy noted in the Mwanza region is comparable to observations made in other sub-Saharan African countries [13]. This is due to limited opportunity for specialty training in neurology [13]. It can also be aggravated by lack of interest in this specialty among doctors.

HCWs in Mwanza region, did not classify patients´ epileptic seizures. This might have led PWE to be treated with similar AEDs, regardless of their seizure types. While a number of epileptic seizure types including partial and generalized tonic clinic seizures can be effectively managed using PB [14] or PHT or CBZ [15], the drugs of choice for managing absence seizures are ethosuxamide [16] and VPA [17]. Levetiracetam, lamotrigine and topiramate can be used as adjunctive therapy for the treatment of partial or generalized seizure. The lack of proper classification of seizures and thus use of the same type of drugs across the patients may be attributed to a lack of adequate epilepsy knowledge among the practitioners or limited choice of available AEDs. In Mwanza, PWE do not have access to the EEG, CT scan and MRI investigations. This is contrary to earlier studies that reported availability of these facilities in more than 70% of African countries [8]. Electroencephalogram is important in confirming the epilepsy diagnosis and classifying epileptic seizures, while CT scan and MRI are useful in establishing the cause of epilepsy. Furthermore, in this study, no cardiac investigations were done to any patient. Electrocardiogram and echocardiogram are useful in excluding arrhythmias and syncope from epileptic seizures. These conditions are commonly mistaken with epilepsy [18,19]. Reasons why PWE in Mwanza region were not fully investigated, include lack of these facilities particularly in the district hospitals. Costs of paying for these investigations could be another reason, particularly for patients who were attended at the consultant referral hospital. Blood tests are important in the follow-up of AEDs side effects. This is because AEDs like PB, PHT and CBZ do alter liver functions [20], serum electrolytes and hematological profile [21-23]. Hematological monitoring is particularly recommended among patients using older AEDs such as PHT, CBZ and VPA [24] since these drugs are more likely to alter hematological profile than the newer AEDs. Despite of blood tests facilities availability in all five hospitals where the present study was done, no follow-up investigations were done in any of the 218 participant patients. This could be due to lack of epilepsy specialists in Mwanza. HCWs managing PWE in the region could have been not aware of importance of these follow-up investigations. Plasma AEDs level monitoring remains a valuable tool in the clinical management of PWE [25], but none of the patient in the present study had his/her plasma AEDs level measured despite some of them having uncontrolled seizures. Anti-epileptic drugs monitoring for selected patients is useful in minimizing AEDs side effects and maximizing seizure control [25]. Selective and appropriate use of AEDs monitoring can lead to better patient care and can significantly enhance the quality of life of patients with epilepsy [25]. The reasons for not practicing therapeutic AEDs monitoring, among others, could be lack of facilities for measuring AEDs, costs associated with it, or a lack of awareness of its importance among practitioners.

Although counseling and follow-up are crucial for medication compliance, PWE in Mwanza were not adequately counseled and followed up. Choosing correct AEDs and compliance to prescribed AEDs are important in successful seizure control. Despite of many side effects, PB and PHT were found to be the most commonly used AEDs. The two drugs are the cheapest amongst the AEDs and this could be one of the reasons for their likely use. For instance, the defined daily dose of other AEDs like CBZ and VPA are 11 times and 16 times that of PB respectively [8]. The two drugs are also in the Tanzanian list of essential drugs. Limited choice and accessibility to AEDs were caused by limited ability of patients to buy the drugs, and drugs availability in the hospital or in the market. Inconsistence use of AEDs and switching from one AED to another followed inconstant drug supply and patients´ inability to afford the costs. In district hospitals AEDs were given free of charge and when the patient´s drugs were not available in the hospital, they were given an alternative available AED. When both PHT and PB were not available, patients were required to buy the drugs from pharmacies. Unfortunately, some patients could not afford to buy the drugs and therefore failed to continue with medication until the hospital was re-supplied with the drugs. Under-medication despite seizure recurrence which was observed in the present study is comparable with a study done in Northern Tanzania where 24.6% of PWE were receiving sub-therapeutic doses of AEDs [6]. The use of sub-therapeutic doses among PWE is likely to be caused by prescribers who are not primarily trained to manage epilepsy. Most of prescribers identified in the present studies were nurses, clinical officers and general medical doctors instead of neurologists or other specialists. Discrepancies between hospitals and practitioners regarding AED withdraw is in line with previous reports which showed a lack of evidence to guide the timing of AED withdrawal in seizure-free adults [26,27]. However, it is generally agreed that it is best to stop AED medications because of their side effects. For children with partial seizures or abnormal EEGs, it is recommended to withdraw AED medications after a period of at least two years or more of seizures-free [26]. Discontinuation of AEDs for PWE after a long period of seizure-free improves neuropsychological performance [28]. Discrepancies between hospitals and practitioners regarding AED withdrawal in this region could be due to lack of common teaching practice regarding epilepsy management.

## Conclusion

The present study shows that, there are no epilepsy specialists in Mwanza region. This led PWE to be diagnosed and treated by nurses, general medical officers and clinical officers. PWE in the studied region were not thoroughly investigated, counseled and followed-up, and had limited choice and accessibility to AEDs. Some patients particularly in district hospitals were under-medicated despite seizure recurrence. There was discrepancy between hospitals and practitioners regarding AED withdrawal. Thus, there is a need for proper and continuous training of the HCWs involved in the management of PWE. In light of the findings of the present study, we recommend Government to supply EEG machines to all district hospitals and CT scan and MRI machines to the referral hospitals. EEG is important for confirming epilepsy diagnosis while CT scan and MRI are useful in exploring the cause of epileptic seizures. We also do recommend initiation and continuous short term training of general medical officers and clinical officers on epilepsy diagnosis and management. In addition to short term training of HCWs who routinely manage PWE, general medical officers should be encouraged for specialization in neurology. Furthermore, the Government should ensure continuous supply of enough and different types of AEDs in all facilities where PWE are attended.

**Funding:** this study was supported by the Fogarty International Center of the National Institutes of Health under Award Number D43TW010138.

### What is known about this topic

- Epilepsy successful treatment depends on appropriate diagnosis, correct choice of AEDs and adherence to the prescribed AEDs;
- There are few specialists (neurologists) to manage PWE in developing countries.

### What this study adds

- PWE in the studies region receive limited care. They are not thoroughly investigated, counseled and followed-up, and had limited choice and accessibility to AEDs;
- There is discrepancy between hospitals and practitioners regarding AED withdrawal;
- Simple intervention measures are recommended to be taken to improve management of PWE in the region.
